# Talking with Actionbits—A Part-Enhanced VLM for Action and Interaction Recognition in Animals

**DOI:** 10.3390/s26061969

**Published:** 2026-03-21

**Authors:** Yang Yang, Ren Nakagawa, Risa Shinoda, Hiroaki Santo, Kenji Oyama, Takenao Ohkawa, Fumio Okura

**Affiliations:** 1 Graduate School of Information Science and Technology, The University of Osaka, Osaka 565-0871, Japan; yang.yang@ist.osaka-u.ac.jp (Y.Y.); rshinoda@iis.u-tokyo.ac.jp (R.S.); 2 Graduate School of System Informatics, Kobe University, Kobe 657-8501, Japanohkawa@kobe-u.ac.jp (T.O.); 3 Institute of Industrial Science, The University of Tokyo, Tokyo 153-8505, Japan; 4 D3 Center, The University of Osaka, Osaka 565-0871, Japan; 5 Graduate School of Agricultural Science, Kobe University, Kobe 657-8501, Japan

**Keywords:** motion analysis, animal interaction, video understanding

## Abstract

Understanding animal actions and interactions is essential for behavior analysis and ecological monitoring. Although large-scale in-the-wild datasets have advanced animal action recognition, existing methods still struggle with fine-grained motion, spatial relations, and multi-individual interactions. To address these challenges, we introduce AIRA, a unified framework for Action and Interaction Recognition in Animals. Built upon a vision–language model (VLM), AIRA learns in an action-centered representation space defined by body parts and their corresponding motions, thereby improving robustness to background noise and enabling cross-species generalization via a unified mammal-centric part ontology. To model actions, we treat body parts and motion as primary cues and introduce **Actionbit** tokens—compact representations for parts and motions generated by a large language model (LLM) that encode which parts move and how. We further propose Part-Enhanced Prompt Fine-tuning (PEPF) to make the VLM explicitly sensitive to part and pose cues. Within PEPF, the Action–actionbit Alignment (AbA) module enriches action representations with fine-grained part–motion semantics, and Part-Vision Prompting (PVP) extracts keyframes through action-aware prompting. Experiments across multiple benchmarks show consistent improvements in both action and interaction recognition, highlighting the importance of action-centered adaptation and relational reasoning for understanding animal behavior in the wild.

## 1. Introduction

Understanding animal actions and interactions is crucial for ecology, animal welfare, and livestock management. Automated behavior recognition enables large-scale, continuous monitoring and early detection of events such as estrus, aggression, or illness.

Prior studies have explored video-based pipelines for wildlife observation [1,2,3], livestock management [4,5,6,7,8], and behavioral research [9,10]. Most approaches encode short clips using 3D CNNs [11,12] or transformer-based video backbones [1,3], sometimes enhanced with pose cues [5,13]. However, these models are trained on limited-scale datasets and often focus on a single species or a constrained environment. Even recent livestock-oriented works [5,8] that explicitly target interaction recognition typically consider only a small set of interaction categories and model interactions within the same species, leaving in-the-wild scenarios with more diverse and complex interactions inadequately addressed.

Recent progress in large-scale unconstrained animal video understanding, driven by large-scale datasets such as Animal Kingdom [14], MammalNet [15], and LoTE-Animal [16], has made it feasible to train vision models on multi-species, multi-behavior videos. While early benchmarks focused on single-animal actions (e.g., feeding, walking), newer datasets include higher-level social interactions (nursing, hunting), highlighting interaction understanding as a key next step.

In parallel, vision–language models (VLMs) such as CLIP [17] have enabled open-vocabulary recognition via text supervision, and extensions like Animal-CLIP [18] have been applied to animal behavior. However, their generalization to animal actions and interactions remains limited due to three challenges: (1) Weak domain knowledge: Current models rely on appearance rather than action semantics, limiting cross-species transfer. (2) Noisy in-the-wild videos: Clutter, occlusion, and long uninformative segments dilute action cues. (3) Limited visual adaptation: Frozen backbones lack sensitivity to part–motion cues crucial for fine-grained recognition.

**Figure 1:**
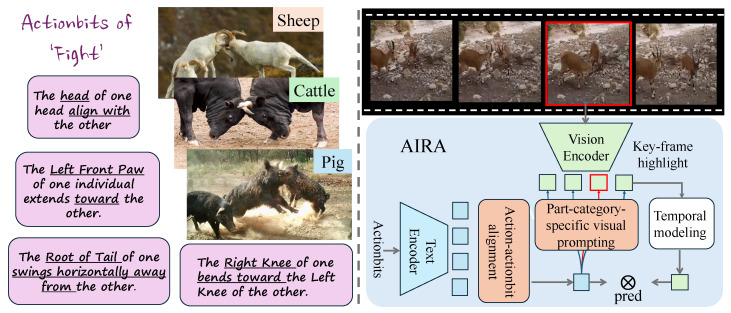
Our VLM-based framework for Action and Interaction Recognition in Animals (**AIRA**) achieves cross-species generalization by learning part–motion semantics shared across animals. To achieve this goal, we introduce **Actionbits**, special tokens that represent fine-grained parts and motions (underlined in the figure). We align part-aware behaviors such as *tail* and *swing* across different animal species through Actionbits, enabling robust action and interaction recognition in the wild.

To address these issues, we propose AIRA, as shown in Figure 1, a unified framework for Action and Interaction Recognition in Animals, which augments pretrained VLMs with part-enhanced perception and strong keyframe localization ability, enabling the learning of action-centered representations that are robust to noisy in-the-wild videos and transferable across mammalian species (i.e., unseen-species generalization) in mammal-dominant benchmarks.

Our key approach is to enrich domain knowledge using automatically generated prompts from large language models (LLMs). We are motivated by the observation that many animal actions and interactions are determined by a small set of visible body parts and their poses/contacts, and often occur in only a few key frames; relying on global appearance or background can therefore lead to shortcut correlations in noisy in-the-wild videos. Specifically, we introduce **Actionbit** tokens—part-centric action descriptions specifying visible body parts, poses, and motion—and complementary animal descriptions that reduce intra-class variation across species. Before fine-tuning, we pre-adapt the CLIP visual encoder using these Actionbits via a lightweight CLIP-Adapter, encouraging sensitivity to part and motion cues while preserving general visual knowledge. To effectively translate the part-informed knowledge into both spatial and temporal evidence, on top of the adapted backbone, we introduce two key modules: (1) Action–actionbit Alignment (AbA), aggregating part-centric prompts into enhanced action embeddings through cross-attention; and (2) Part-category-specific Visual Prompting (PVP), which uses these embeddings to reweight frame-level features, localizing keyframes, and suppressing irrelevant frames. Species-level descriptions further serve as priors to stabilize recognition across diverse animals.

### Contributions

In summary, different from generic prompt learning or parameter-efficient fine-tuning that mainly adapts VLMs with general prompts, AIRA injects part-grounded external knowledge and enforces part-level semantic consistency to support compositional action reasoning. It unifies part-aware representation learning, parameter-efficient visual adaptation, and semantics-guided keyframe localization, advancing fine-grained action and interaction recognition in the wild, involving the following chief contributions:

- We propose AIRA, a part-enhanced prompt-tuning framework for in-the-wild animal action recognition that leverages frozen CLIP backbones and automatically generated action/animal descriptions, removing the need for expert annotations.
- We introduce Actionbits, which are used for adaptation to enhance the CLIP visual encoder’s sensitivity to body-part locations, poses, and motion patterns, thereby improving the recognition of fine-grained animal actions.
- We design PEPF, which features AbA for aligning part-centric Actionbits and PVP for part-aware keyframe localization, thereby enabling explicit sensitivity to body parts and poses in in-the-wild videos.

## 2. Related Work

### 2.1. Animal Visual Recognition

Visual recognition of animals has long been a central task in ecological computer vision, supporting applications such as biodiversity monitoring, conservation, and species identification. Early research focused on species-level classification, supported by fine-grained visual datasets such as Caltech-UCSD Birds [19], NABirds [20], and iNaturalist [21]. These benchmarks enabled the development of fine-grained recognition models such as Bilinear CNNs (B-CNN) [22], MAMC [23], and TransFG [24], which capture subtle appearance variations among closely related species. More recent datasets, including the iNat2021 and iNat2023 challenges, extended this line of work to long-tailed, real-world species distributions.

In addition to species recognition, pose estimation has become a crucial component of visual animal understanding, providing both geometric and behavioral cues across species. Datasets such as Animal Pose [25] and AP-10K [26] introduced large-scale multi-species keypoint annotations, promoting generalizable pose estimation methods beyond humans. Recent vision–language approaches, including BioCLIP [27] and BioCLIP 2 [28], further extended open-vocabulary recognition to the animal domain, enabling flexible, zero-shot identification of new species and attributes. While these studies have advanced our understanding of appearance at a superficial level, they primarily focus on static images and lack modeling of temporal motion or social interactions, which are central to behavioral analysis.

### 2.2. Animal Action Recognition

Recognizing animal actions and interactions extends beyond static appearance to temporal dynamics and relational reasoning, aiming to capture behaviors such as locomotion, feeding, and social interactions. Early work adapted human action recognition architectures, such as C3D [29], I3D [30], SlowFast [31], X3D [32], and MViT [33], to wildlife videos, but these models struggled to generalize due to inter-species variation and the scarcity of labeled data.

The release of large-scale datasets such as Animal Kingdom [14], MammalNet [15], and LoTE-Animal [16] has enabled supervised learning for multi-species behavior understanding, covering both individual actions (e.g., feeding, walking) and interactions (e.g., nursing, hunting). These benchmarks established action recognition as a key foundation for ecological monitoring and cross-species analysis. Several works have also developed animal-focused behavior recognition systems [5,6,8,34] tailored to specific species and deployment settings, such as livestock monitoring (e.g., understanding cattle behavior and interactions in farm environments). These approaches are effective in their target domains, yet often operate in closed or controlled settings or focus on a single species, limiting their scalability to open-world, multi-species behavior recognition. Although beyond the vision community, recent research has incorporated acoustic modalities to enhance understanding of behavior. Bioacoustic models such as BirdNET [35], BioLingual [36], and NatureLM-Audio [37] use deep audio or multimodal embeddings to recognize species and their vocal behaviors. Efforts like SSW60 [38] and ImageBind [39] explore joint representations of images, audio, and text, suggesting that multimodal fusion can yield richer animal behavior representations.

Recently, Animal-CLIP [18] extended VLMs for open-vocabulary animal action recognition, aligning videos with natural-language action descriptions. However, such models rely heavily on global appearance and lack explicit understanding of body parts, pose dynamics, and inter-animal relations that drive complex behaviors. Recent methods introduce pose-aware or part-level modeling [26], but they still require strong supervision or well-annotated keypoints, which are impractical in the wild. More broadly, recent VLM adaptations often rely on a single class prompt or a small set of learned prompt tokens, which provides limited structure for capturing part-centric behavioral cues.

Our work builds upon these trends by proposing a part-enhanced vision–language framework that injects structural priors (body parts and pose cues) and keyframe localization into pretrained VLMs. By decomposing each interaction into two individual actions and a relational description, we enable cross-species generalization and robust understanding of fine-grained animal interactions in natural environments.

## 3. Method: Action and Interaction Recognition in Animals (AIRA)

Our design is driven by a simple observation: animal behaviors are more stable in terms of ‘what body parts move and how’ than in global appearance. In in-the-wild videos, the same action may occur across species with very different textures, shapes, and backgrounds, while the discriminative signal often concentrates on a few part–motion cues (e.g., head orientation, foreleg contact, tail swing). Off-the-shelf VLMs and standard video pooling tend to over-rely on holistic appearance or background co-occurrence, which hurts cross-species transfer and tail-class recognition.

**Figure 2:**
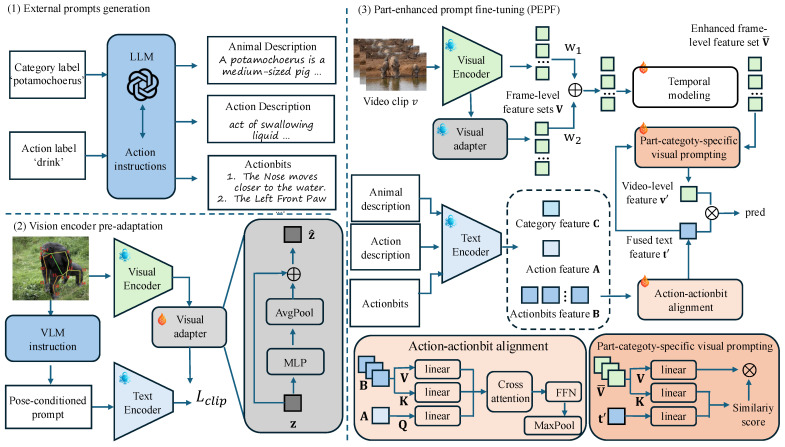
**Method overview.** (1) External prompt generation. Given human instructions and action labels, an LLM produces animal descriptions, action descriptions, and part-centric Actionbits that encode visible body parts, poses, and motion states. (2) Vision encoder pre-adaptation. A frozen CLIP image–text encoder is equipped with a lightweight visual adapter and trained with pose-conditioned prompts using a CLIP loss, making the visual backbone more sensitive to part and motion cues while preserving its original representation space. (3) Part-Enhanced Prompt Fine-tuning (PEPF). Video frames are encoded by the adapted visual encoder and processed by a temporal module; Action–actionbit Alignment refines the action text with Actionbits and animal descriptions, while Part-category-specific Visual Prompting uses the enhanced action feature to reweight frame-level features and highlight keyframes. The final prediction is obtained by computing the similarity between the video-level feature $v^{\prime }$ and fused text feature $t^{\prime }$ followed by softmax.

Our framework has three key components, as illustrated in Figure 2. (1) *External knowledge generation* (Section 3.1). We automatically construct animal/action texts and Actionbits using LLM/VLMs to inject missing domain knowledge for robust, cross-species behavior understanding. (2) *Vision encoder pre-adaptation* (Section 3.2). We adapt a pretrained CLIP vision backbone with Actionbits supervision to make vision features explicitly sensitive to body parts and motion cues. (3) *Part-Enhanced Prompt Fine-tuning (PEPF)* (Section 3.3). Motivated by the limitations of off-the-shelf VLMs, which often rely on coarse action names on the text side and holistic appearance correlations on the vision side, we introduce two complementary modules to distill and localize part–motion evidence. On the text branch, Action–actionbit Alignment (AbA) aligns each action category with the most discriminative body-part relations encoded in Actionbits, yielding a refined action embedding. On the vision branch, Part-category-specific Visual Prompting (PVP) leverages Actionbit prompts as semantic guidance to emphasize salient part-related regions and suppress irrelevant context, thereby enabling more robust recognition in cluttered and cross-species settings.

### 3.1. External Prompts Generation

Following prior work, we construct two types of external prompts, *animal descriptions* and *action descriptions*, to inject semantic priors about species and behaviors. Unlike narrative free-form text, we strictly constrain generation to a *part–motion* action space to avoid noise from scenes, intents, or physical phenomena. We adopt a unified mammal-centric part ontology and restrict all descriptions to this shared vocabulary (species-level masking removes parts that do not exist). We encode discriminative cues with concise *Actionbits* (pose-like part–motion atoms) that specify *who moves* and *how*.

We design separate constrained templates for animals and actions. The allowable tokens are drawn only from {body part, motion, optional direction/intensity, optional contact target}; terms about environment (e.g., “pond”), intent (e.g., “to hydrate”), or non-visual behavior (e.g., “growling”) are explicitly denied. For non-mammal species, the part vocabulary is pruned by a species dictionary before decoding. In addition, we set the constraints during the prompt generation as follows:

- Produce 10 Actionbits per action label.
- Limit body-part vocabulary to the 17 AP-10k terms.
- Do not include intent or subjective language.
- Describe actions from a fixed, mid-range observation.

We use the GPT-5 model for the constrained prompt generation and match the pretrained CLIP text encoder’s maximum sequence length by limiting prompts to 77 tokens.

**Figure 3:**
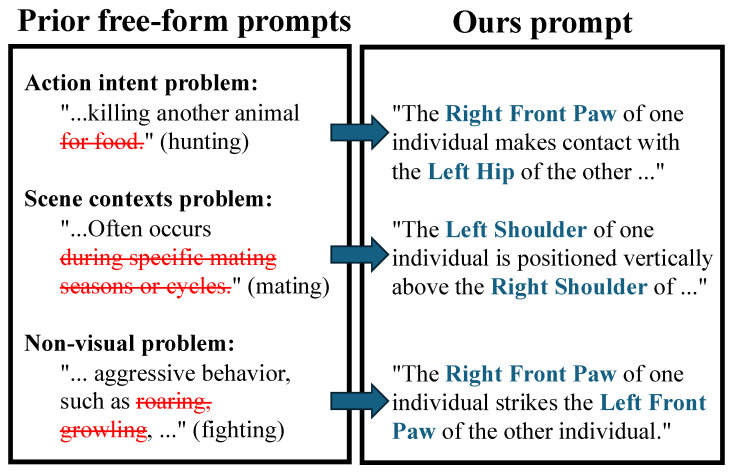
**Comparison of prompt samples**. (**Left**): Ordinary free-form prompts generated by LLMs often contain noise like intent, scene, or non-visual phrases (red strike-through). (**Right**): Our prompts focus on visible body parts and their spatial relations (blue text).

We here compare our part-enhanced prompts with ordinary free-form prompts in Figure 3. Appendix A provides the complete prompt list for better reproducibility.

### 3.2. Vision Encoder Pre-Adaptation

Pre-adapting the vision branch aims to correct a central limitation we observed in off-the-shelf CLIP: it is weakly sensitive to *animal parts* and *part-driven motion cues*, which later prevents it from aligning well with the external prompts introduced in Section 3.1. To address this with minimal computational requirements and without disrupting CLIP’s text semantics, we freeze the text encoder and adapt only the vision branch using parameter-efficient LoRA [40], inserting low-rank adapters into the vision linear layers. This approach preserves the base weights and keeps the number of trainable parameters small.

#### 3.2.1. Pretraining Data Preparation

**Figure 4:**
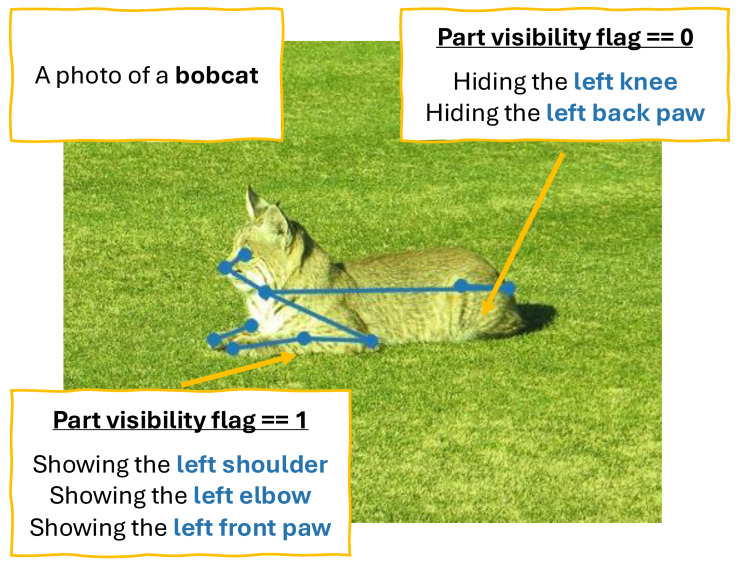
**Sample of training data generated for CLIP vision encoder pre-adaptation**. For each image, we automatically generated dense, part-centric prompts.

To fine-tune CLIP, we built a pretraining set from the AP-10K dataset [26], which contains 10,015 images from 54 animal species with skeleton keypoint annotations. We converted the skeleton data into CLIP-ready semantic language information, as shown in Figure 4. Specifically, using a simple rule-based method, we classified visible and occluded joints and converted them into sentences along with animal species information.

#### 3.2.2. Adaptation

Inspired by the CLIP-Adapter architecture [41], we fine-tune CLIP in a parameter-efficient manner by leaving the pretrained image and text encoders $e_{v}\left(\cdot \right)$ and $e_{t}\left(\cdot \right)$ frozen and attaching a lightweight adapter on top of the visual branch.

Let $e_{v}\left(\cdot \right)$ and $e_{t}\left(\cdot \right)$ denote the pretrained CLIP image and text encoders with parameters $\theta_{v}$ and $\theta_{t}$, respectively. Given an input image (frame) *I*, we first extract the CLIP feature

(1) $$z=e_{v}\left(I;\theta_{v}\right)\in R^{d}.$$

We then feed z into a shallow adapter network $f_{v}\left(\cdot |\psi \right)$ parameterized by ψ, implemented as a two-layer MLP with a nonlinear activation:

(2) $$f_{v}\left(z|\psi \right)=W_{2}\;\sigma \left(W_{1}z\right),$$

where $W_{1}$ and $W_{2}$ are learnable weights and σ(·) denotes a point-wise nonlinearity (e.g., GELU [42]). The adapted visual feature is obtained via a residual connection,

(3) $$\hat{z}=z+f_{v}\left(z|\psi \right),$$

which preserves the original CLIP representation while allowing the adapter to capture domain-specific cues. We denote the resulting adapted image encoder as

(4) $${\hat{e}}_{v}\left(I\right)=\hat{z}.$$

On the text branch, we directly use the frozen CLIP text encoder $e_{t}\left(\cdot ;\theta_{t}\right)$ without additional adapters, since text descriptions already reside in a relatively clean and stable semantic space. During this adaptation stage, we optimize only the adapter parameters ψ with a CLIP-style symmetric contrastive loss, using image–text pairs from our animal-action corpus. Given a batch of images ${\left\{I_{i}\right\}}_{i=1}^{N}$ and their corresponding textual descriptions ${\left\{y_{i}\right\}}_{i=1}^{N}$, we compute

(5) $${\tilde{v}}_{i}={\hat{e}}_{v}\left(I_{i}\right),\;{\tilde{t}}_{i}=e_{t}\left(y_{i};\theta_{t}\right),$$

and train the adapter with the same symmetric CLIP loss applied to the pairs $\left({\tilde{v}}_{i},{\tilde{t}}_{i}\right)$ as

(6) $$\begin{array}{cc} L_{\mathrm{CLIP}}\; & =\frac{1}{2}\left(L_{I\to T}+L_{T\to I}\right)\\ \; & =\frac{1}{2}\left(\frac{1}{N}\sum_{i=1}^{N}-\log\frac{\exp(\mathrm{sim}\left({\tilde{v}}_{i},{\tilde{t}}_{i}\right)/\tau )}{\sum_{j=1}^{N}\exp\left(\mathrm{sim}\left({\tilde{v}}_{i},{\tilde{t}}_{j}\right)/\tau \right)}+\frac{1}{N}\sum_{i=1}^{N}-\log\frac{\exp(\mathrm{sim}\left({\tilde{t}}_{i},{\tilde{v}}_{i}\right)/\tau )}{\sum_{j=1}^{N}\exp\left(\mathrm{sim}\left({\tilde{t}}_{i},{\tilde{v}}_{j}\right)/\tau \right)}\right), \end{array}$$

where *N* and τ represent the number of samples per batch and learnable temperature parameter, respectively. After this stage, we fix ${\hat{e}}_{v}$ and $e_{t}$, and use the adapted visual features as the input to the Part-Enhanced Prompt Fine-tuning described in Section 3.3. In this way, the visual branch used in later stages inherently contains both the original global CLIP semantics and the domain-adapted, part-sensitive cues provided by the adapter.

### 3.3. Part-Enhanced Prompt Fine-Tuning (PEPF)

The final fine-tuning stage is designed to overcome two remaining bottlenecks of off-the-shelf VLM-based video recognition: (i) coarse text prompts provide limited part–motion evidence for fine-grained actions, and (ii) noise frames hide discriminative cues when features are uniformly pooled or when generic temporal attention overfits to background shortcuts. To overcome these problems, we leverage the external knowledge (Section 3.1) and the pre-adapted vision encoder (Section 3.2) to fine-tune the model in a strictly action-related space. On the vision branch, we encode video frames with part-enhanced adapter ${\hat{e}}_{v}$. These frame-level features are passed to a temporal learning module, which captures spatio-temporal features, yielding a video-level representation. On the text branch, we obtain embeddings for both the animal descriptions and the action descriptions using the frozen CLIP text encoder $e_{t}$. We then introduce two learnable components: aba (AbA), which strengthens the action semantics by integrating the action label with its set of action bits via cross-attention, and Part-category-specific Visual Prompting (PVP) that utilizes the refined action-category-centered textual feature to guide the visual branch toward salient frames.

Specifically, given a video clip $v_{i}=\left\{I_{i,1},\ldots ,I_{i,n}\right\}$, we first obtain per–frame image embeddings $v_{i,j}\in V_{i}$ from the vision encoder. To consider the temporal relation between frames, following prior video–language frameworks (e.g., XCLIP [43]), we inject temporal order by adding a learnable temporal positional embedding $P\in R^{n\times E}$ and feed the frame feature sequence $V_{i}$ into a small temporal Transformer with 3 layers, denoted as $e_{\mathrm{temp}}\left(\cdot |\rho \right)$:

(7) $${\bar{V}}_{i}\;=e_{\mathrm{temp}}\left(V_{i}\right),$$

where ${\bar{V}}_{i}=\left[v_{i,1},\ldots ,v_{i,n}\right]$ are the fine-grained (frame-level) visual features for the video, ρ is the number of learnable parameters and *n* is the number of frames. In contrast to previous methods that simply average all frame-level features to obtain the video representation, we introduce a Part-specific Visual Prompting (PVP) that leverages action semantics to identify salient frames and reweight them for aggregation, thereby obtaining the video-level feature $v_{i}^{\prime }\in R^{d}$.

Simultaneously, given the action label $A_{i}$, its *K* actionbits $B_{i}=\left\{b_{i,1},\ldots ,b_{i,K}\right\}$, and animal description $C_{i}$, we apply $e_{t}$ to get initial action textual features $A\in R^{d}$, Actionbit textual features $B\in R^{d\times K}$, and animal textual features $C\in R^{d}$. Denoting the AbA module as $e_{\mathrm{aba}}\left(\cdot |\varphi \right)$, we obtain an enhanced feature $A^{\prime }\in R^{d}$ that aggregates evidence from the Actionbits to highlight determinative parts and motions of the action. Concretely, $A^{\prime }\in R^{d}$ can be represented as

(8) $$A^{\prime }=e_{\mathrm{aba}}\left(A,B|\varphi \right),$$

where φ refers to learnable parameters.

We then integrate species knowledge, following the strategy introduced in [18], by conditioning the enhanced action feature $A^{\prime }$ on the animal descriptions C through an action-conditioned prompting function $e_{\mathrm{ac}}\left(\cdot |\phi \right)$. Concretely, we feed $A^{\prime }$ and the corresponding animal description into $e_{\mathrm{ac}}$ to obtain the final textual feature $t_{i}^{\prime }$, which injects species-specific semantics into the action representation while reusing a similar cross-attention-based design (see [18] for more details).

During training, the frozen CLIP backbones provide the base image/text spaces, while we only optimize a small set of parameters, ϕ, φ, and ρ. Given the batch pairs ${\left\{\left(v_{i}^{\prime },t_{i}^{\prime }\right)\right\}}_{i=1}^{N}$, we also optimize the symmetric CLIP loss similar to Equation (6), which can be formulated as

(9) $$\begin{array}{cc} L_{\mathrm{CLIP}}\; & =\frac{1}{2}\left(L_{V\to T}+L_{T\to V}\right)\\ \; & =\frac{1}{2}\left(\frac{1}{N}\sum_{n=1}^{N}-\log\frac{\exp(\mathrm{sim}\left(v_{i}^{\prime },t_{i}^{\prime }\right)/\tau )}{\sum_{j=1}^{N}\exp\left(\mathrm{sim}\left(v_{i}^{\prime },t_{j}^{\prime }\right)/\tau \right)}+\frac{1}{N}\sum_{i=1}^{N}-\log\frac{\exp(\mathrm{sim}\left(t_{i}^{\prime },v_{i}^{\prime }\right)/\tau )}{\sum_{j=1}^{N}\exp\left(\mathrm{sim}\left(t_{i}^{\prime },v_{j}^{\prime }\right)/\tau \right)}\right). \end{array}$$

#### 3.3.1. Action–Actionbit Alignment (AbA)

AbA is a novel mechanism designed to enrich action representations with fine-grained part–motion cues. Compared to normal attention, AbA makes the subsequent alignment with part-enhanced visual features more discriminative via a consistency check. Given the action embedding $A\in R^{d}$ and its *K* Actionbit textual features $B={\left[b_{1},\ldots ,b_{K}\right]}^{⊤}\in R^{K\times d}$, we use multi-head cross-attention, which is inspired by [44], that builds a two-branch model with Transformer architecture. We let the action embedding be the Query *Q* to query the part–motion tokens. Formally, let $W_{k}$, $W_{q}$ and $W_{v}$ be the projection matrices which project A to query space and project B to key and value space, respectively:

(10) $$Q=AW_{q},K=BW_{k},V=BW_{v}.$$

Then, the output of the attention layer is calculated by

(11) $$\mathrm{Attention}\left(Q,K,V\right)=\mathrm{softmax}\left(\frac{QK^{T}}{\sqrt{d_{k}}}\right)V,$$

where $d_{k}$ refers to the dimension of the feature.

Multi-head attention breaks down the input (Queries, Keys, and Values) into several smaller, parallel segments. Each segment independently focuses on a different aspect or part of the input sequence. The results from these independent focuses are then combined and synthesized to create a richer, more comprehensive final output, allowing the model to simultaneously capture diverse relationships between actions and actionbits. The multi-head output can be computed by

(12) $$\mathrm{MultiHead}\left(Q,K,V\right)=\mathrm{Concat}\left(h_{1},\ldots ,h_{i}\right)W^{0},$$

where head $h_{i}$ is an attention layer described in Equation (11), and weight matrix $W^{0}$ is combined as a linear transformation to produce the final output of the multi-head attention layer. We also add a standard residual path and layer normalization around attention, followed by a position-wise feed-forward network as

(13) $$\begin{array}{cc} \bar{A} & =\mathrm{LN}(B+\mathrm{MultiHead}\left(AWq,BW_{k},BW_{v}\right)), \end{array}$$

(14) $$\begin{array}{cc} \bar{A} & =\mathrm{LN}(\bar{A}+\mathrm{FFN}\left(\bar{A}\right)), \end{array}$$

where LN(·) refers to layer normalization [45]. We then aggregate with max pooling over the all of the Actionbits to obtain a single enhanced action feature $A^{\prime }$ as

(15) $$A^{\prime }={\mathrm{MaxPool}}_{k=1,\ldots ,K}\left({\bar{A}}_{k}\right).$$

#### 3.3.2. Part-Category-Specific Visual Prompting (PVP)

While AbA injects fine-grained part–motion cues into the action representation, the visual stream still suffers from noisy frames in in-the-wild videos, such as severe occlusions or background distractors. Since normal attention can be distracted by background or irrelevant regions, we introduce Part-category-specific Visual Prompting (PVP) that restricts each part feature to the corresponding part evidence by uses the enhanced action feature $A^{\prime }$ as a semantic query to highlight key frames and suppress irrelevant ones. Concretely, given the frame-level features ${\bar{V}}_{i}=\left[v_{i,1},\ldots ,v_{i,n}\right]$, similar to AbA, we first project the enhanced action feature and the frame features into a shared attention space:

(16) $$Q_{v}=A^{\prime }W_{q}^{v},\;K_{v}={\bar{V}}_{i}W_{k}^{v},\;V_{v}={\bar{V}}_{i}W_{v}^{v},$$

where $W_{q}^{v}$, $W_{k}^{v}$, and $W_{v}^{v}$ are learnable projection matrices. We then compute the similarity between $A^{\prime }$ and each frame via scaled dot-product attention to obtain frame-wise importance scores:

(17) $$\alpha =\mathrm{softmax}\;\left(\frac{Q_{v}K_{v}^{⊤}}{\sqrt{d_{k}}}\right)\in R^{n},$$

where the *t*-th entry $\alpha_{t}$ reflects how compatible frame *t* is with the part–category text query. These scores are used as weights to aggregate all frame-level features into a keyframe-weighted video-level feature as the final visual representation $v^{\prime }$:

(18) $$v^{\prime }=\alpha \;V_{v}\in R^{d}.$$

### 3.4. Zero-Shot Inference

At test time, given an input video *v* and a set of candidate action–category labels, we kept all parameters of AIRA fixed and reused the same pipeline as in training. If an action or animal category was not included in the fine-tuning set, its action description and Actionbits were automatically generated according to our prompting rules, and its animal description was produced by a VLM and then encoded by the CLIP text encoder, without requiring any additional training. Zero-shot prediction is finally obtained by computing similarities between $v^{\prime }$ and all candidate $t^{\prime }$ and selecting the label with max similarity as

(19) $$\begin{array}{cc} \hat{y} & =\underset{j\in \{1,\ldots ,C\}}{arg max}p\left(j|v^{\prime }\right), \end{array}$$

(20) $$\begin{array}{cc} p(j|v^{\prime }) & =\frac{\exp(\mathrm{sim}\left(v^{\prime },t_{j}^{\prime }\right)/\tau )}{\sum_{c=1}^{C}\exp\left(\mathrm{sim}\left(v^{\prime },t_{c}^{\prime }\right)/\tau \right)}. \end{array}$$

## 4. Experiments

We evaluated our approach on two large-scale, in-the-wild animal behavior benchmarks and report results under comparable protocols.

### 4.1. Experimental Setup

#### 4.1.1. Datasets

We used three representative datasets for comparison. **MammalNet** [15] is a large-scale video benchmark of higher-level mammalian behaviors collected from documentaries and public web sources across diverse habitats. It includes approximately 173 mammal species and 12 behavior classes (e.g., hunting, nursing, migrating). We follow the original protocol and use an 8:2 train–test split. We also report the performances using the **LoTE-Animal** [16] dataset, focusing on endangered species and real-world field footage with frequent occlusion, motion blur, and sensor noise. The dataset covers 11 species and 21 action categories, making it suitable for assessing robustness and generalization to new species. We adopt the official split for training and evaluation. **Animal Kingdom** [14] is a diverse, multi-species benchmark for animal action understanding. It spans major animal groups (e.g., mammals, birds, insects, marine life) and $N_{\mathrm{actions}}$ action categories, captured across varied environments.

#### 4.1.2. Metrics

We evaluate the models on single-label MammalNet and LoTE-Animal using Top-k accuracy (Top-1 and Top-5) and mAP at the CLIP level. Results are additionally summarized over head/middle/tail frequency groups to evaluate long-tail robustness. All metrics are computed on the official test sets.

#### 4.1.3. Baselines

We compare our method with representative vision-only action recognition architectures, I3D [30], X3D [32], and MViT [33]. Besides, as VLMs close to ours, we use VideoPrompt [46], EVL [47], Text4Vis [48], and Animal-CLIP [18]. For VLM baselines, we follow the common setup with frozen CLIP vision and text encoders. All methods are trained under matched protocols with the same input resolution, frame sampling, and optimizer. We finetune each model following the settings reported in their original papers on MammalNet. For Animal-CLIP, we reproduce its strategy by generating species and action descriptions using the prompts reported in the original paper.

#### 4.1.4. Implementation Details

For the pre-adaptation, We use CLIP ViT-B/16 as the backbone and perform a lightweight pre-adaptation stage before the main fine-tuning. In this stage, we insert a bottleneck CLIP-Adapter into the CLIP visual encoder and optimize it using the Actionbit-based video–text objective. The CLIP-Adapter follows a bottleneck design with reduction ratio 4 and dropout 0.1. For each training sample, we randomly draw $N_{\mathrm{caption}}\;=\;5$ candidate Actionbit prompts from the available prompt pool and use them to form the text set for contrastive learning.

We then use the pre-adopted CLIP as the backbone architecture for finetuning. For each video, we extract *n* frames, which are then cropped to a size of 224×224. We trained with AdamW with a learning rate of $8\times 10^{-6}$, weight decay of $10^{-3}$, cosine schedule for 30 epochs with five 5 warm-up epochs, batch size of 64 with a gradient accumulation of 16, a dropout of 0.5, and a softmax head. At test time, we adopted 12-view inference (4 clips × 3 crops).

### 4.2. Results

#### 4.2.1. Quantitative Comparison

Table 1 reports the top-one and top-five accuracies (%) across the MammalNet dataset and LoTE dataset. As shown, our AIRA model mostly achieves the best accuracy among state-of-the-art vision-based and VLM-based action recognition methods. Vision models designed for human action recognition, including MViT [33], struggle to handle animal actions due to the inherent diversity and sparsity of animal action datasets. VLMs, especially Animal-CLIP [18], yield relatively accurate estimates by leveraging animal-specific vision–language information. Notably, Animal-CLIP attains particularly high top-one accuracy on MammalNet partly because the dataset covers many species and some rare behaviors are strongly correlated with specific animal categories. In this setting, prompting with the ground-truth animal label provides a powerful prior that can significantly narrow down the plausible action space. In contrast, AIRA does not rely on animal labels at inference time, yet achieves comparable top-1 accuracy while substantially improving top-5 performance. This suggests that our action-bit-based training better captures pose–action cues beyond category shortcuts and is better suited to generalizing to unseen animal species where reliable category labels may be unavailable.

Table 2 reports top-one/top-five accuracies (%) on the LoTE-Animal dataset under the protocol of Section 4.1, with head/middle/tail breakdowns. Vision models tailored for human actions struggle with LoTE’s in-the-wild footage, which is characterized by heavy occlusion, motion blur, and sensor noise, leading to higher confusion among fine-grained animal behaviors. VLM baselines benefit from language priors, with Animal-CLIP performing strongly, yet they remain sensitive to background bias and species appearance shifts. By contrast, AIRA leverages action-guided training and part-category-specific prompting to strengthen pose/motion cues, yielding larger gains on tail classes and clips with severe occlusion.

**Figure 5:**
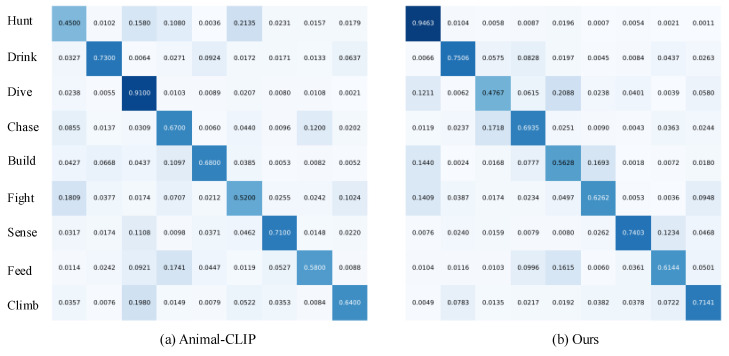
Confusion matrices on MammalNet dataset for (**a**) Animal-CLIP and (**b**) our model.

To further analyze fine-grained confusions, we visualize the confusion matrix of AIRA and AnimalCLIP as shown in Figure 5. Compared with Animal-CLIP, our method increases the diagonal values for most classes, especially for visually complex behaviors (e.g., ‘Hunt’, ‘Fight’).

#### 4.2.2. Evaluation of Generalization Ability

To further evaluate the generalization ability of our model, we followed the experimental protocol of Animal-CLIP on the Animal Kingdom dataset [14]. Specifically, we selected 15 common mammal species, of which 10 were seen during training and the remaining 5 were unseen species. We considered 14 common action categories for evaluation. As shown in Table 3, our method achieves the strongest transfer performance and shows particularly clear improvements on unseen species.

#### 4.2.3. Ablation Study

We conducted ablation studies to assess the contribution of each component in our framework. Since the major components in our method are (1) use of external prompts, (2) the vision encoder pre-adaptation step, and (3) AbA and (4) PVP, the key modules in PEPF, we ablate each from our full model. Table 4 and Table 5 summarize the results on the MammelNet and LoTE datasets. We confirm that each module contributes to the superior accuracy of our AIRA framework.

## 5. Conclusions

This paper introduced AIRA, a part-enhanced vision–language framework for recognizing animal actions and interactions in challenging real-world settings. AIRA is built on the premise that understanding animal behavior requires reasoning over body-part motion, which is far more consistent across species than raw appearance. To this end, we augment pretrained VLMs with Actionbits, compact part-centric motion descriptors automatically generated by a large language model, and integrate them through Part-Enhanced Prompt Fine-tuning (PEPF). PEPF combines Action–actionbit Alignment (AbA), which enriches action embeddings with fine-grained part–motion semantics, and Part-aware Visual Prompting (PVP), which highlights key frames and suppresses background distractions. Together, these components produce action-centered representations that are robust to noise, clutter, and occlusion while maintaining sensitivity to subtle body-part configurations.

Experiments on the MammalNet dataset demonstrate that AIRA consistently improves performance in both single-action and interaction recognition. These results demonstrate the effectiveness of injecting structured, part-based priors into vision–language models and illustrate the promise of motion-centered, cross-species representations for understanding ecological behavior. By shifting emphasis from appearance to shared anatomical and behavioral cues, AIRA moves toward more reliable, generalizable recognition of animal behavior in the wild.

### Limitations

AIRA relies on LLM-generated Actionbits, which may be incomplete for rare species or subtle behaviors, and currently assumes a mammal-centric part ontology that limits applicability to other taxa. In addition, PVP emphasizes short-term cues and does not fully capture long-range temporal structure. Future work will explore extending AIRA to broader species groups, richer multimodal cues, and modeling of longer behavioral sequences. 

## Figures and Tables

**Table 1:** **Comparison on MammalNet**. Top-5 and top-1 accuracies (%) are shown. Results are reported for head/middle/tail frequency groups, as well as overall. The best accuracies are highlighted **bold**.

Methods	Category	Top-5 Accuracy (%)				Top-1 Accuracy (%)			
		Head	Medium	Tail	Overall	Head	Medium	Tail	Overall
I3D [30]	Vision	70.31	54.10	44.26	57.06	46.3	35.0	14.8	32.1
X3D [32]	Vision	77.35	60.47	47.48	62.68	36.0	15.9	32.2	38.0
MViT [33]	Vision	72.53	56.58	46.89	59.49	50.9	42.4	20.0	37.8
VideoPrompt [46]	VLM	77.98	57.62	47.73	62.11	54.6	44.7	23.4	39.2
EVL [47]	VLM	77.35	60.47	47.48	61.09	53.2	44.1	22.8	37.7
Text4Vis [48]	VLM	79.23	72.61	62.10	71.77	64.5	37.9	40.2	45.6
Animal-CLIP [18]	VLM	83.90	75.46	65.73	75.55	67.71	41.37	**43.73**	**50.94**
AIRA (Ours)	VLM	**96.45**	**93.38**	**76.90**	**88.91**	**70.78**	**47.49**	41.79	**52.65**

**Table 2:** **Comparison on LoTE**. Top-5 and top-1 accuracies (%) are shown. Results are reported for head/middle/tail frequency groups, as well as overall. The best accuracies are highlighted **bold**.

Methods	Category	Top-5 Accuracy (%)				Top-1 Accuracy (%)			
		Head	Medium	Tail	Overall	Head	Medium	Tail	Overall
I3D [30]	Vision	98.39	92.67	38.96	95.03	84.00	83.76	47.56	81.94
X3D [32]	Vision	98.64	93.28	40.12	97.54	86.01	84.37	47.56	83.58
MViT [33]	Vision	99.47	95.67	42.33	94.32	75.11	74.75	25.77	72.33
VideoPrompt [46]	VLM	95.71	92.23	40.57	92.88	71.67	45.56	11.59	61.52
EVL [47]	VLM	98.85	93.11	40.87	96.73	72.47	67.34	25.00	68.52
Text4Vis [48]	VLM	97.55	93.02	41.25	96.89	77.51	60.15	20.73	69.84
Animal-CLIP [18]	VLM	99.86	**95.53**	42.75	97.99	86.20	**85.66**	43.90	83.47
AIRA (Ours)	VLM	**99.90**	94.79	**43.93**	**98.63**	**89.90**	84.92	**43.93**	**84.19**

**Table 3:** Generalization ability comparison on seen and unseen animals. ${\mathrm{mAP}}_{S}$ and ${\mathrm{mAP}}_{U}$ denote mAP on seen and unseen animal categories, respectively, and Hm is their harmonic mean. The best accuracies are highlighted **bold**.

Methods	${\mathrm{mAP}}_{S}$	${\mathrm{mAP}}_{U}$	Hm
I3D [30]	43.47	18.17	25.63
X3D [32]	46.80	24.98	32.57
MViT [33]	38.31	17.01	23.56
VideoPrompt [46]	60.73	18.15	27.95
EVL [47]	58.61	20.14	29.98
Text4Vis [48]	42.76	30.47	35.58
Animal-CLIP [18]	61.33	33.41	43.26
AIRA (Ours)	**61.74**	**35.71**	**46.59**

**Table 4:** **Ablation study of our components on MammalNet dataset.** Ablations of external prompts, vision encoder pre-adaptation, Action–actionbit Alignment (AbA), and Part-category-specific Visual Prompting (PVP). The best accuracies are highlighted **bold**.

Methods	Top-5 Accuracy (%)				Top-1 Accuracy (%)			
	Head	Medium	Tail	Overall	Head	Medium	Tail	Overall
W/o external prompts	95.61	82.33	56.98	79.30	62.18	40.71	33.56	41.78
W/o vision encoder pre-adaptation	89.19	69.51	68.32	76.48	68.34	45.28	39.18	49.26
W/o Action–actionbit Alignment (AbA)	95.96	75.88	61.47	77.6	66.21	45.62	37.45	48.28
W/o Part-category-specific Visual Prompting (PVP)	93.26	71.35	55.96	73.3	63.71	42.33	37.28	47.21
Ours (full model)	**96.45**	**93.38**	**76.90**	**88.91**	**70.78**	**47.49**	**41.79**	**52.65**

**Table 5:** **Ablation study of our components on LoTE dataset.** Ablations of external prompts, vision encoder pre-adaptation, Action–actionbit Alignment (AbA), and Part-category-specific Visual Prompting (PVP). The best accuracies are highlighted **bold**.

Methods	Top-5 Accuracy (%)				Top-1 Accuracy (%)			
	Head	Medium	Tail	Overall	Head	Medium	Tail	Overall
W/o external prompts	95.52	91.17	39.23	94.27	77.56	70.17	35.69	76.76
W/o vision encoder pre-adaptation	97.26	93.16	42.86	96.55	83.72	82.29	42.97	82.19
W/o Action–actionbit Alignment (AbA)	99.74	94.52	43.65	97.75	87.34	**85.15**	42.55	83.22
W/o Part-category-specific Visual Prompting (PVP)	98.71	93.63	**44.01**	94.85	83.68	82.89	43.56	82.15
Ours (full model)	**99.90**	**94.79**	43.93	**98.63**	**89.90**	84.92	**43.93**	**84.19**

## Data Availability

The code of this research will be published publicly on GitHub at https://github.com/Yrainy0615/Animal-Interaction.git.

## Abbreviations

**Abbrev.**	**Meaning**
AIRA	Action and Interaction Recognition in Animals (our framework)
VLM	Vision–Language Model
Actionbit	Part-centric textual primitive describing visible body parts, pose/contact, and motion cues.
PEPF	Part-Enhanced Prompt Fine-tuning
AbA	Action–actionbit Alignment
PVP	Part-category-specific Visual Prompting

## Appendix A. More Details About External Prompts

To provide a detailed view of the generated external prompts, we present examples for animal description and Actionbits in Table A1. Our external prompts comprise (i) *animal descriptions* that capture morphology, typical postures, and habitat priors, and (ii) *actionbits*, a compact set of motion/part cues.

**Table A1:** External prompts of animal description and actionbits.

**Action**	
**Drinking Water**	*The Neck lowers towards the water surface. The Nose moves closer to the water. The Left Front Paw remains stationary. The Right Front Paw remains stationary. The Neck moves slightly in the vertical direction. The Nose touches the water surface. The Left Shoulder maintains a stable position. The Right Shoulder maintains a stable position. The Neck raises slightly after contact with water. The Root of Tail remains stationary.*
**Sleep**	*The Left Eye remains closed. The Right Eye remains closed. The Nose is stationary. The Neck is in a relaxed position. The Root of Tail is motionless. The Left Shoulder is at rest. The Right Shoulder is at rest. The Left Front Paw is in a relaxed position. The Right Front Paw is in a relaxed position.*
**Fight**	*The Nose of one individual moves toward the Neck of the other individual. The Left Front Paw of one individual extends toward the Right Back Paw of the other individual. The Right Eye of one individual aligns with the Left Eye of the other individual. The Left Shoulder of one individual advances in the horizontal direction toward the Right Shoulder of the other individual. The Right Front Paw of one individual makes contact with the Left Hip of the other individual. The Neck of one individual lowers below the Neck of the other individual. The Left Back Paw of one individual moves rapidly in the horizontal direction toward the Right Back Paw of the other individual. The Right Knee of one individual bends toward the Left Knee of the other individual. The Root of Tail of one individual aligns vertically with the Left Hip of the other individual. The Left Elbow of one individual maintains proximity to the Right Elbow of the other individual.*
**Hunt**	*The Neck moves rapidly downward in the vertical direction. The Nose points downward sharply. The Left Shoulder and Right Shoulder move slightly forward. The Left Elbow and Right Elbow bend slightly. The Left Front Paw and Right Front Paw remain stationary. The Neck jerks backward slightly. The Nose moves slightly upward. The Left Hip and Right Hip remain stable. The Root of Tail remains stationary. The Neck returns to a neutral position.*
**Animal**	
**Panthera**	*A panthera is a large cat with a muscular body, short fur, and a sleek appearance. It typically has a black or dark brown coat with distinctive spots or rosettes. Panthers have strong jaws, sharp teeth, and keen eyesight for hunting.*
**Leptailurus**	*Leptailurus is a genus of small to medium-sized wild cats. They typically have a slender body, short legs, a short face with rounded ears, and a long tail. Their coat can vary in color from sandy yellow to reddish-brown with distinctive spots or rosettes. A Leptailurus can be identified by its physical characteristics and markings.*
**Acinonyx**	*An acinonyx, commonly known as a cheetah, is a large, slender big cat with a distinctive spotted coat ranging from golden yellow to pale tan. It has a small, rounded head with black tear-like facial markings, a long and sleek body, and long legs built for speed. Cheetahs are known for their black spots on their bodies and the sleek.*
**Vulpes**	*Vulpes is a genus of small to medium-sized foxes. They typically have a slender body, pointed muzzle, large ears, long bushy tail, and fur that ranges in color from red to gray. Vulpes are known for their intelligence and agility, making them skilled hunters and adaptable to various environments.*
